# Enhancing colorectal cancer histology diagnosis using modified deep neural networks optimizer

**DOI:** 10.1038/s41598-024-69193-x

**Published:** 2024-08-22

**Authors:** Reham Elshamy, Osama Abu-Elnasr, Mohamed Elhoseny, Samir Elmougy

**Affiliations:** 1 Department of Computer Science, Faculty of Computers and Information, Mansoura University, Mansoura, 35516 Egypt; 2 Department of Information Systems, Faculty of Computers and Information, Mansoura University, Mansoura, 35516 Egypt

**Keywords:** Adagrad optimizer, Adam optimizer, Fine-tuning technique, Transfer learning technique, Colorectal cancer histology, Computer science, Computational science

## Abstract

Optimizers are the bottleneck of the training process of any Convolutionolution neural networks (CNN) model. One of the critical steps when work on CNN model is choosing the optimal optimizer to solve a specific problem. Recent challenge in nowadays researches is building new versions of traditional CNN optimizers that can work more efficient than the traditional optimizers. Therefore, this work proposes a novel enhanced version of Adagrad optimizer called SAdagrad that avoids the drawbacks of Adagrad optimizer in dealing with tuning the learning rate value for each step of the training process. In order to evaluate SAdagrad, this paper builds a CNN model that combines a fine- tuning technique and a weight decay technique together. It trains the proposed CNN model on Kather colorectal cancer histology dataset which is one of the most challenging datasets in recent researches of Diagnose of Colorectal Cancer (CRC). In fact, recently, there have been plenty of deep learning models achieving successful results with regard to CRC classification experiments. However, the enhancement of these models remains challenging. To train our proposed model, a learning transfer process, which is adopted from a pre-complicated defined model is applied to the proposed model and combined it with a regularization technique that helps in avoiding overfitting. The experimental results show that SAdagrad reaches a remarkable accuracy (98%), when compared with Adaptive momentum optimizer (Adam) and Adagrad optimizer. The experiments also reveal that the proposed model has a more stable training and testing processes, can reduce the overfitting problem in multiple epochs and can achieve a higher accuracy compared with previous researches on Diagnosis CRC using the same Kather colorectal cancer histology dataset.

## Introduction

Optimizers are essential in modifying neural networks’ parameters so that they can produce accurate estimations or evaluations. The main goal of optimizers is to incrementally adapt the model's parameters in a way that reduces the gap between expected results and actual desired outcomes. Recent challenges are trying to produce enhanced versions of traditional optimizer’s algorithms that can work faster and more efficient than the traditional optimizers. This work introduces a novel enhanced version (SAdagrad) of one of the most popular optimizers in deep learning (Adagrad) that avoid the main drawbacks of Adagrad optimizer in vanishing the value of learning rate and modifying its parameters during the training process. In this work Adam and Adagrad optimizers are used in the comparison of SAdagrad’s results.

There are many critical parameters in learning process one of them is learning rate parameter which is also known as step size. Choosing the optimal value of step size for each iteration in the learning process is very important step in speeding the training process and enhances the overall performance of the optimizer. Adagrad optimizer can’t choose the optimal value of learning rate for each iteration when increasing the time of training process. In order to compare the performance of SAdagrad, Adam and Adagrad optimizers, this paper build CNN model using one of the challenging CRC dataset which is divided into different types of colorectal cancer images. CRC is a form of cancer which impacts the large intestine, also known as the colon, or the rectum. It is sometimes referred to it as bowel cancer, colon cancer or rectal cancer. It can develop when abnormal cells in the outer layer of the rectum proliferate excessively^1^.

Colorectal cancer is the third most common kind of cancer in the globe^2^. Moreover, colorectal cancer affects both men and women equally. Actually, CRC can happen at any age, although it typically affects those over the age of 50. Because the initial phases of colorectal cancer typically have no indications, regular examination is critical for early identification. Advanced colorectal cancer indications include fluctuations in bowel routines, pain in the abdomen, bloating, blood in the stool, and abnormal reduction in weight. In colorectal cancer patients, surgery to surgically eliminate the malignant tumor is often adhered to by radiation or chemo therapy to eliminate the remaining cancerous cells^3^. While in clinical situations, visual inspection of histopathology plates is still essential, malignant tissue can be examined rapidly and quantitatively using digital analysis of images. Using automated procedures to help conserve time and limit the possibility of mistaken assumptions has become critical. Lately, plenty of researches have been put into developing artificial intelligence to be used for diagnosing various cancer types. Moreover, numerous sorts of optimizers have been used in a few of these efforts in CNN. By embedded optimization execution, extra hyper parameters are introduced to Adam's main ones, preserving the gradient direction in^4^. This paper introduces SAdagrad optimizer as an improved version of the Adagrad^5^ algorithm; it is SAdagrad optimizer, which can avoid the lack of tuning learning rate values. Adagrad optimizer helps adapt the learning rate^6^ value for each Step in the training process, which is different from SGD^7^ or Nesterov^8^ accelerated gradient optimizers that use a fixed value of learning Step—rates for all epochs. The proposed training model combines our modified SAdagrad optimizer with a transfer learning technique, such as a pre-trained model (MobileNet) with fine-tuning technique, to avoid overfitting and enhance the overall performance of the SAdagrad optimizer on the colorectal cancer dataset.

The paper is organized as follows: section "Main contribution and highlights" provides main contribution and highlights, section "Literature review" provides Literature review, section "The proposed modified optimizer (SAdagrad)" discusses proposed optimizer (SAdagrad), section "Experimental Results and evaluations" elucidates experimental results and evaluations, section "Conclusion" presents the conclusion, section "Limitations and future directions:" clarifies the results limitations and future directions and Sect. 8 clarifies the availability of data and materials.

## Main contribution and highlights

- In this paper, we present a novel optimization algorithm SAdagrad which designed to overcome the limitations inherent in the Adagrad optimizer. Adagrad, while effective in certain contexts, suffers from several drawbacks that can hinder its performance, including:
- Monotonically Decreasing Learning Rates: Adagrad's reliance on accumulating squared gradients leads to a monotonically decreasing learning rate over time.
- Lack of Momentum: Unlike some modern optimizers, such as Adam or RMSprop, Adagrad does not incorporate momentum terms to accelerate convergence. We designed CNN model that can compete models under Adam optimizer with momentum.
- In this paper we present CNN model that combine the learning transfer learning technique with Weight Decay Technique.
- Evaluation on Colorectal Cancer Histology Diagnosis Dataset CRC: The proposed model's efficacy is evaluated using the challenging Colorectal Cancer Histology Diagnosis dataset, a standard benchmark in medical image analysis. The dataset's complexity allows thorough examination of the model's generalization and resilience.
- Significant Performance Improvement: Experimental findings illustrate a substantial performance boost of the proposed model compared to baseline methods and existing state-of-the-art models. The enhanced Adagrad optimizer contributes to higher accuracy, accelerated convergence, and enhanced robustness.

## Literature review

### Background

**Figure 1:**
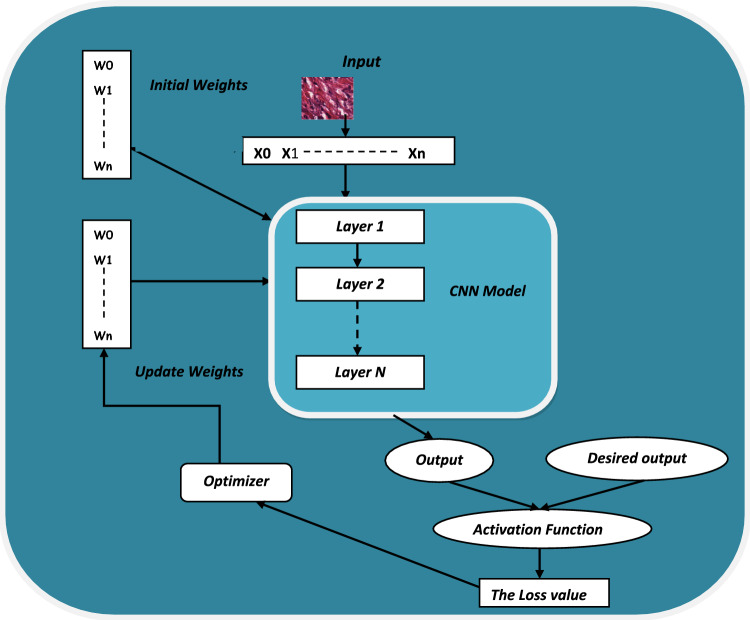
The training process of CNN model.

This section concentrates on the concept of the way deep neural network optimizers operate and shows some research that suggested ways to improve a variety of standard optimizers. Firstly, it discusses some of the well-known optimizers that are employed in several of deep learning challenges especially on classification of CRC dataset. The main goal of any CNN model is to find the most accurate values of its parameters which reduce the gap between the output result of the model and the desired result; Fig. 1 shows this process. There are many types of optimizers in deep learning regards to how they update the model’s parameters. This work will focus on Adagrad and Adam optimizers. Adagrad refers to a category of optimization algorithms used in deep learning models. These algorithms aim to efficiently update the weights of a model during the training process by tuning the learning rate value for each step. Traditional gradient-based optimization methods, such as SGD^9^, use a fixed learning rate for all parameters. However, this approach can lead to slow Convolutionergence or oscillation in the training process. Adaptive gradient-based algorithms address this issue by individually adapting the learning rates based on the historical gradients of each parameter. One of the most popular optimizers in this class is Adagrad^10^. Adagrad modulates the learning speed for every period according to the corresponding square root of the total number of squared slopes over a period of time. This technique efficiently reduces the learning rate for often modified variables while increasing it for seldom altered ones. The main spot of Adagrad is to change the step size of each parameter with respect to the square root of the sum of squared gradients for that parameter. This implies that learning steps will be significantly reduced for parameters with large gradients, whereas parameters with small gradients will undergo more substantial updates. The update equation for Adagrad for parameter θ at time Step t is as shown in Eq. (1).

1 $${\theta }_{t+1}={\theta }_{t}-\frac{\eta }{\sqrt{{s}_{t}+\epsilon }} {\text{g}}_{\text{t}}$$

where η is the learning step for each instance and S is the squared of decay average at time t. Another important optimizer in this class is Adam optimizer; the next sub-section clarifies how it works.

Adam optimizer^11^ is a widely used optimization technique for deep learning. It was introduced by Diederik, Kingma and Jimmy Ba in 2014. The basic idea behinds Adam algorithm is to compute learning step using information from the gradients' first and second moments compute for each parameter. As shown in Eqs. (2) and (3). In traditional SGD^12^, the learning rate is fixed and is the same for all parameters. This can lead to slow Convolutionergence or getting stuck in local minima. Furthermore, Eq. (4) gives the final step of updating θ^13^.

2 $$ \mu_{t}^{ \wedge } = \frac{{\mu_{t} }}{{1 - \beta_{t}^{1} }} $$

3 $$ v_{t}^{ \wedge } = \frac{{v_{t} }}{{1 - \beta_{t}^{2} }} $$

4 $${\theta }_{t+1}={\theta }_{t}-\frac{\eta }{\sqrt{{v}_{t}+\epsilon }} {\mu }_{t}$$

Adam optimizer computes the first and second moments of the gradients using exponential moving averages, which gives more weight to recent gradients. It specifically maintains track of the squared magnitudes and the exponentially decaying average of previous gradients. With larger changes for small slope values and lower changes for large slope variables, these two moments are then utilized to determine an adaptive learning rate for each parameter. To account for their initialization at zero, Adam additionally incorporates bias corrections to the first and second moment estimations. This is crucial, particularly in the early stages of training when estimations are incorrect. The next sub-section focuses on related works.

### Related works

For the purpose of categorizing and grading colorectal cancer tissues, CNN models have been developed and evaluated using the Kather colorectal cancer histology dataset^14^. Many researchers have been able to evaluate the effectiveness of various models and pinpoint effective strategies for raising the precision and dependability of CRC detection. A publicly accessible collection, which is called the Kather colorectal cancer histology dataset, contains 15,000 histopathology shots of colorectal cancer samples labeled with the related clinical outcomes. In their work, Kather et al.^15^ have introduced the colorectal cancer dataset. Since its publication, this dataset has received a lot of attention from scientists who have utilized it to generate and evaluate CNN models to determine the types of colorectal cancer tissues. Janowczyk and Madabhushi^16^ have used Kather colorectal cancer dataset to train and test a several CNN models using AlexNet^17^, VGGNet^18^ and GoogLeNet^19^.

They have reported an overall accuracy of 79%. Cruz-Roa et al.^20^ Have also used the CRC dataset to train and evaluate a CNN model for the task of detecting invasive ductal carcinoma in breast cancer histology images. They have achieved 93% accuracy with their model. Moreover, Wang et al.^21^ have used the CRC dataset to train an attention-based CNN model for the task of grading colorectal cancer histology images; their model achieved accuracy of 88%. In^22^ Rizalputri et al. have utilized various methodologies, such as K-Nearest Neighbor^23^ CNN, Random Forest, and Logistic Regression, to analyze the Kather colon histological dataset. They have aimed to evaluate the effectiveness of each technique and determine the optimal algorithm. Based on their findings, CNN is proved to be the most successful method, achieving an accuracy rate of 82.2%. In^24^ have suggested a model adopting Kather colorectal cancer histological assessment to automatically identify eight tissues seen in CRC evaluation. Additionally, it has been implemented to transfer knowledge based on CNN structures by them. CNNs' structure has been adjusted in order to extract information from images and input them into machine learning techniques such as k-Nearest Neighbours, Multilayer Perceptrons, Random Forest, Naive Bayes, and Support Vector Machines (SVM). The best performance has been achieved by DenseNet169 with SVM (RBF), with a level of accuracy of 92.08%. In^25^ have introduced a technique for determining histology pictures' great geometric variety by extracting four local characteristics: local architectural information, local geometric information, local energetic information, and local patterns.

These features are obtained using the Riesz transform and monogenic local binary patterns. They tested their method on two multiclass histology picture datasets (Kather and Kimiapath 24) and obtained a categorization and getting accuracy of 90% on each. Numerous studies have concentrated on the application of transfer learning in CNN models. For instance, in reference^26^, a deep learning model incorporating transfer learning and attention mechanisms has been devised for estimating electromyography hand gestures. This model comprises a feature extraction system with focus modules for extracting pertinent features, a three-layer fully interconnected label classifier, and a gesture estimator employing a threshold voting algorithm to provide gesture estimation results even before the completion of the hand motion. In^27^, the framework of optimization, the highlighted research provides a succinct introduction to the newly developed idea of TL approaches for a collection of functions. The authors underline the need to address the problem of where to transfer in addition to the three crucial ones of what, how, and when to move. It is stated and explained that the first stage in transfer learning is to consider the issue, "From where to transfer?”. In^28^, the authors devised an approach for categorizing CRC tissues using multi-spectral HI. They identified three different kinds of tissue related with CRC varieties, namely Benign Hyperplasia (BH), Intraepithelial Neoplasia (IN), and carcinoma. In^29^, the authors proposed a reliable CAD method for Metastatic Lymph Nodes (LNM) in CRC by combining HI analysis and feature assessment. To recognize CRC tissue using different data sets, the researchers created a deep learning model based on the CNN structure. In^30^ the authors presented a novel machine learning framework designed for the diagnosis of brain diseases. Specifically, the authors introduce a Projective Parameter Transfer-based Sparse Multiple Empirical Kernel Learning Machine. This approach incorporates advanced computational techniques to analyze complex brain imaging data. By leveraging sparse multiple empirical kernels and projective parameter transfer, the method aims to enhance diagnostic accuracy and efficiency in identifying various brain disorders. The research contributes to the development of innovative tools for medical imaging analysis, potentially leading to improved diagnostic capabilities and treatment outcomes in the field of neuroscience.

In^31^ delves into the evolving landscape of utilizing artificial intelligence (AI) in cancer diagnosis and therapy. It may cover a wide range of topics, including: current applications: exploring how AI is currently being applied in cancer diagnosis, such as in image analysis for early detection, pathology interpretation, and radiology. Therapeutic approaches: discussing the role of AI in developing personalized treatment plans, including precision medicine approaches based on genomic analysis and predictive modeling. Challenges and limitations: addressing the challenges and limitations of AI in cancer diagnosis and therapy, such as data quality issues, interpretability of AI algorithms, and ethical considerations. Future perspectives: providing insights into the future directions and potential advancements in AI technologies for cancer management, including the integration of AI into clinical practice, advancements in AI algorithms, and emerging research areas. Overall, the paper likely aims to provide a comprehensive overview of the current state-of-the-art, challenges, and future prospects of AI in cancer diagnosis and therapy, offering valuable insights for researchers, clinicians, and policymakers in the field. In^32^ this paper presented a novel deep learning architecture, termed HADCNet, and designed for the automatic segmentation of COVID-19 infections in medical imaging data, such as chest X-rays or CT scans. The method likely utilizes a combination of attention mechanisms, dense connections, and dilated convolutions to accurately identify and segment regions of COVID-19 infection within medical images. This research may contribute to the development of computer-aided diagnostic tools for COVID-19 detection and monitoring. In^33^ this paper presented a novel deep learning approach for image dehazing, which is the process of enhancing visibility and removing haze or fog from images. The proposed method likely utilizes multi-level fusion techniques and attention mechanisms within a convolution neural network (CNN) architecture to effectively address the challenges associated with image dehazing. This research may contribute to advancements in image processing techniques for improving the quality of images captured in hazy or foggy conditions. The next section clarifies our proposed enhanced of optimizer SAdagrad algorithm. In^34^ this paper proposes an efficient image decolonization method using a multimodal contrast-preserving measure. By preserving contrast across multiple modalities, the approach achieves effective decolonization while maintaining image fidelity. This contributes to improved image quality and enhances the interpretability of medical imaging data. In^35^ this paper introduces an unsupervised domain adaptation method for medical semantic segmentation. It employs style adaptation and boundary enhancement techniques to adapt the model to new domains without labeled data. The approach enhances segmentation accuracy and generalization across diverse medical imaging datasets, facilitating robust clinical applications. In^36^ the paper presents an advanced evolutionary machine learning approach using a joint self-adaptive simulated annealing algorithm to predict recurrent spontaneous abortion (RSA). This method effectively enhances prediction accuracy by optimizing feature selection and model parameters. The study demonstrates the potential of evolutionary algorithms in improving clinical outcomes for RSA prediction. In^37^ the paper introduces LDANet, a deep learning framework designed for automatic lung parenchyma segmentation from CT images. LDANet combines Convolution neural networks (CNN) with a local distance-aware (LDA) module to enhance segmentation accuracy. The model effectively addresses challenges such as varying lung shapes and pathological abnormalities. Extensive experiments demonstrate that LDANet significantly outperforms existing methods in terms of accuracy and robustness in lung segmentation tasks. In^38^ The paper reviews deep learning methods for medical image fusion, emphasizing their ability to integrate multimodal medical images for enhanced diagnostic accuracy. It categorizes various deep learning approaches, including CNNs and generative adversarial networks (GANs), highlighting their strengths and limitations. The review also discusses the performance of these methods in terms of image quality, computational efficiency, and clinical applicability. The authors conclude with future directions and challenges in advancing medical image fusion through deep learning technologies. In^39^ the paper proposes a bioinspired scene classification method using deep active learning tailored for remote sensing applications. The approach leverages a deep learning framework combined with active learning strategies to enhance the efficiency and accuracy of scene classification. By mimicking biological learning processes, the method selectively queries the most informative data points to improve model training. Extensive experiments demonstrate that this bioinspired method significantly outperforms traditional techniques in terms of classification accuracy and data efficiency. The study highlights the potential of integrating bioinspired mechanisms with deep learning for advanced remote sensing applications. In^40^ the paper presents a novel approach for high-quality retinal vessel segmentation using a generative adversarial network (GAN) with a large receptive field. This method improves segmentation accuracy by effectively capturing complex vessel structures in retinal images. The GAN framework, with its large receptive field, enhances the ability to discern fine details and overall vessel morphology. Experimental results demonstrate that this technique outperforms existing methods in terms of precision and robustness. The study underscores the potential of advanced GAN architectures in medical image analysis, particularly for retinal vessel segmentation. In^41^ They introduced a method for unsupervised domain adaptation in medical semantic segmentation by incorporating style adaptation and boundary enhancement techniques. Style adaptation aligns feature distributions across different domains, ensuring robust performance in varying imaging conditions. Boundary enhancement techniques are employed to improve the delineation of object boundaries, enhancing segmentation accuracy. This approach addresses challenges such as variability in medical imaging data and improves segmentation quality by effectively leveraging unlabeled target domain data. In^42^ they presented a method for detecting epileptic seizures in EEG signals using sparse multi scale radial basis function networks and the Fisher vector approach. This approach involves extracting multi scale features from EEG signals using sparse radial basis function networks, which capture both local and global characteristics of the data. The Fisher vector approach is then applied to encode these features into a fixed-length representation, enhancing discriminative power and reducing the dimensionality of the data. By combining these techniques, the method achieves robust seizure detection by effectively distinguishing seizure events from normal brain activity patterns. This contributes to the development of automated systems for epilepsy diagnosis and monitoring, offering potential improvements in accuracy and efficiency compared to traditional methods.

## The proposed modified optimizer (SAdagrad)

As we all know, the goal of any optimizer is to minimize the loss function, which is the variance between the real result of our structure and the expected result that we expect to accomplish. There are numerous forms of optimizers that can be used to attain the lowest value for the loss function. So, to be able to accomplish this, we need several sorts of optimizers, which can be divided into two categories. The first category is First Order Optimization techniques: these methods apply Gradient values to reduce or increase the Loss function according to parameters. The most popular optimizer in this type is Gradient optimizer and it variations. He the second category is Second-order techniques make use of the second order derivative. This is called Hessian that can be utilized to decrease or enhance the Loss function. The Hessian can be viewed as matrices of second-order partial derivatives. The second derivative is costly to calculate, but it is rarely used. This stage proposes (SAdagrad) an enhanced version of Adagrad (Adaptive Gradient Descent) algorithm which is one of the variation optimizers that further optimize Gradient Descent optimizer. The adaptive gradient descent algorithm is a bit distinct from conventional gradient descent algorithms. This is due to the fact it employs various learning rates for each iteration. The change in learning rate is determined by the parameter differences encountered during training. The more parameters are modified, the less the learning rate varies. This adjustment is extremely useful because real-world datasets contain both sparse and dense features. The Adagrad algorithm uses the below formula in Eqs. (5) and (6) to update the weights.

5 $$ {\text{w}}_{{\text{t}}} = {\text{w}}_{{{\text{t}} - 1}} - {\upeta }_{{\text{t}}}{\prime} \frac{{\partial {\text{L}}}}{{\partial {\text{w}}\left( {{\text{t}} - 1} \right)}} $$

6 $$ {\upeta }_{{\text{t}}}{\prime} = \frac{{\upeta }}{{{\text{sqrt}}\left( {{\upalpha }_{{\text{t}}} + \varepsilon } \right)}} $$

Here the alpha is the various learning steps at, n is a constant, and epsilon is a tiny positive parameter to avoid division by 0.

The main drawback of the Adagrad optimizer is that it drops the learning rate quickly and roughly. There could be a point where the learning rate becomes incredibly low. This is due to the squared gradients in the denominator continued to accumulate, causing the denominator to increase. Due to low learning rates, the model eventually becomes unable to gain new knowledge, compromising its accuracy. So in order to enhance this drawback of Adagrad, this stage proposes an enhanced version of Adagrad algorithm (SAdagrad), where the learning rate gradually shrinks. Because the learning step in the later training phase is so tiny, it must be determined by calculating the initial global learning rate. A procedure to the steps of Adagrad algorithm that manages and schedules the values of learning rate for each epoch in the training process is added to solve these problems with Adagrad. Consequently, this prevents the step size from becoming very small during increasing the total number of periods in training and raises productivity of Adagrad*.*

The Steps of SAdagrad algorithm are presented in detail as given below.
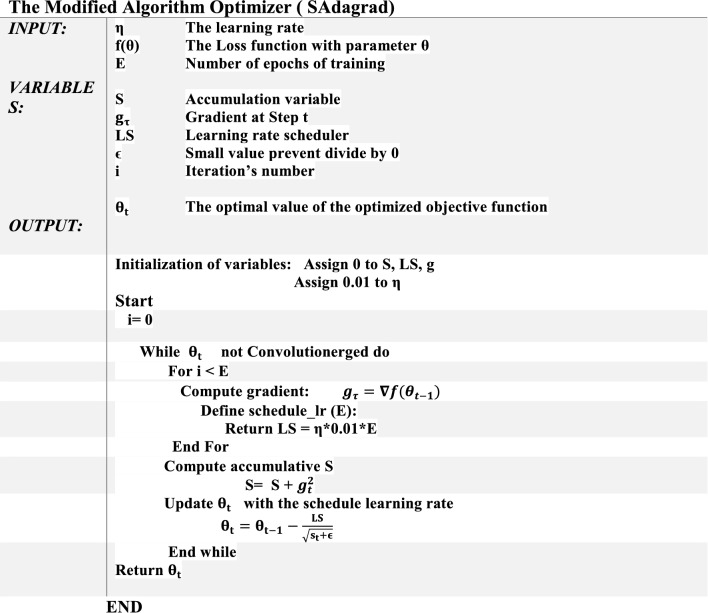


Obviously, SAdagrad algorithm gains its power from managing and scheduling the values of step size for each iteration of the overall process. SAdgrad begins with initial **η** value 0.01, as clarified in Step 1. Then, it starts in Step 4 with computing the gradient of the objective function $({{\varvec{\theta}}}_{{\varvec{t}}})$. Next, in Step 5, it begins to build a schedule of learning rate values LS that aims to manage and adapt the suitable value of step size for each epoch avoiding the drawbacks of the traditional Adagrad optimizer, such as the very small size or giving insufficient value of learning rate. After that, SAdagrad stores all previous square gradients as a history, as clarified in Step 7, which helps it to tune the value of LS for the next iteration. Finally θ is updated using the past of all earlier gradients on timetable of learning rate values, as shown in Step 8.

The key operation in SAdagrad is computing and storing the sum of squared gradients for each parameter. Therefore, the overall complexity per iteration of Adagrad is O (n). So SAdagrad hasn’t increased the complexity of Adagrad optimizer in mathematical operations. It's important to note that while Adagrad's per-iteration complexity is linear with respect to the number of parameters, its storage requirements grow linearly with the number of parameters as well, which can become a limiting factor for very large models. Additionally, the accumulation of squared gradients can lead to diminishing learning rates over time, which we address this issue in SAdagrad optimizer.

## Experimental results and evaluations

CRC dataset and its assessment measures are discussed in this section. Moreover, the experiments of the proposed model with the different three optimizers SAdagrad, Adagrad, and Adam are illustrated.

### Dataset description

Colorectal cancer histology dataset CRC^43^ is an important tool for scientists concerned with creating machine learning algorithms for automated diagnosis and prediction. It is a useful tool for pathology research and therapeutic applications due to its size, diversity, and in-depth annotations. All of the histopathological images of colorectal cancer tissue samples have a pixel size of 0.495 m and a resolution of 150 * 150 px (74 * 74 m). The researchers at the Institute of Pathology at Heidelberg University Hospital in Germany, under the direction of Dr. Jakob Nikolas Kather, have produced the CRC dataset. The dataset is comprised of over 10,000 histological pictures of colorectal cancer tumors from various people. All of them is identified by a special code and includes comprehensive patient data, like the patient's age, gender, tumour stage and colon or rectum where the tumour is located. The dataset has eight distinct cell classes: "TUMOUR", "STROMA", "COMPLEX", "LYMPHO", "DEBRIS", "MUCOSA", "ADIPOSE", and "EMPTY". In the context of a Colorectal Cancer (CRC) dataset, the classes typically represent different types of tissue structures or components found in histopathological images. Here's a description of each class: TUMOUR: This class represents regions within the histopathological images that contain cancerous cells or tumors. These regions are characterized by abnormal cell growth and may exhibit various morphological features indicative of cancer. STROMA: The stroma class refers to the supportive tissue framework surrounding the tumor cells. It includes connective tissue, blood vessels, and other structural elements that provide support to the tumor. COMPLEX: Complex regions in the dataset refer to areas where the tissue structure is intricate or displays mixed characteristics. These regions may contain a combination of tumor cells, stromal elements, and other tissue components. LYMPHO: Lympho class represents lymphocytic infiltration within the tissue. These regions contain immune cells, such as lymphocytes, which are involved in the body's immune response to cancer cells. DEBRIS: Debris class includes areas within the images that contain cellular or extracellular debris. These regions may result from cell death, tissue damage, or other pathological processes. MUCOSA: Mucosa class refers to the epithelial lining of the gastrointestinal tract, particularly the innermost layer called the mucous membrane. It plays a crucial role in protecting and lubricating the digestive tract. ADIPOSE: Adipose class represents adipose tissue or fat cells present within the histopathological images. Adipose tissue serves various functions, including energy storage and insulation. EMPTY: The empty class typically denotes regions within the images that do not contain any significant tissue structures. These areas may represent background noise or artifacts in the images. In fact, the dataset has already been utilized in a number of investigations, including the creation of deep learning algorithms for the automatic detection and classification of tissue features related to CRC.

### The model structure

**Figure 2:**
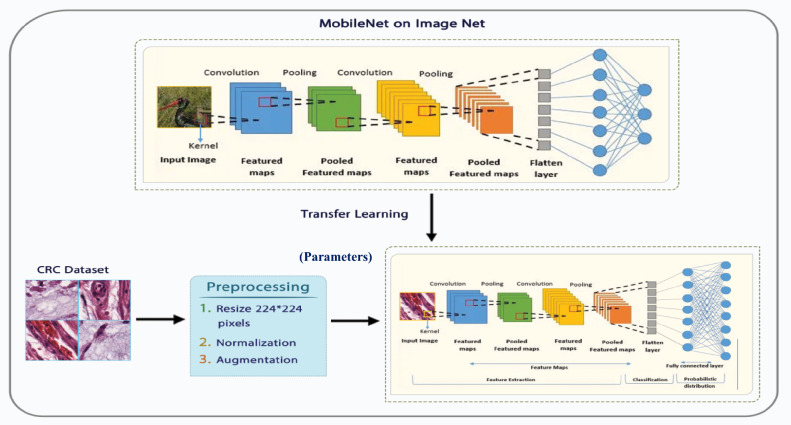
The proposed framework with learning transfer.

**Figure 3:**
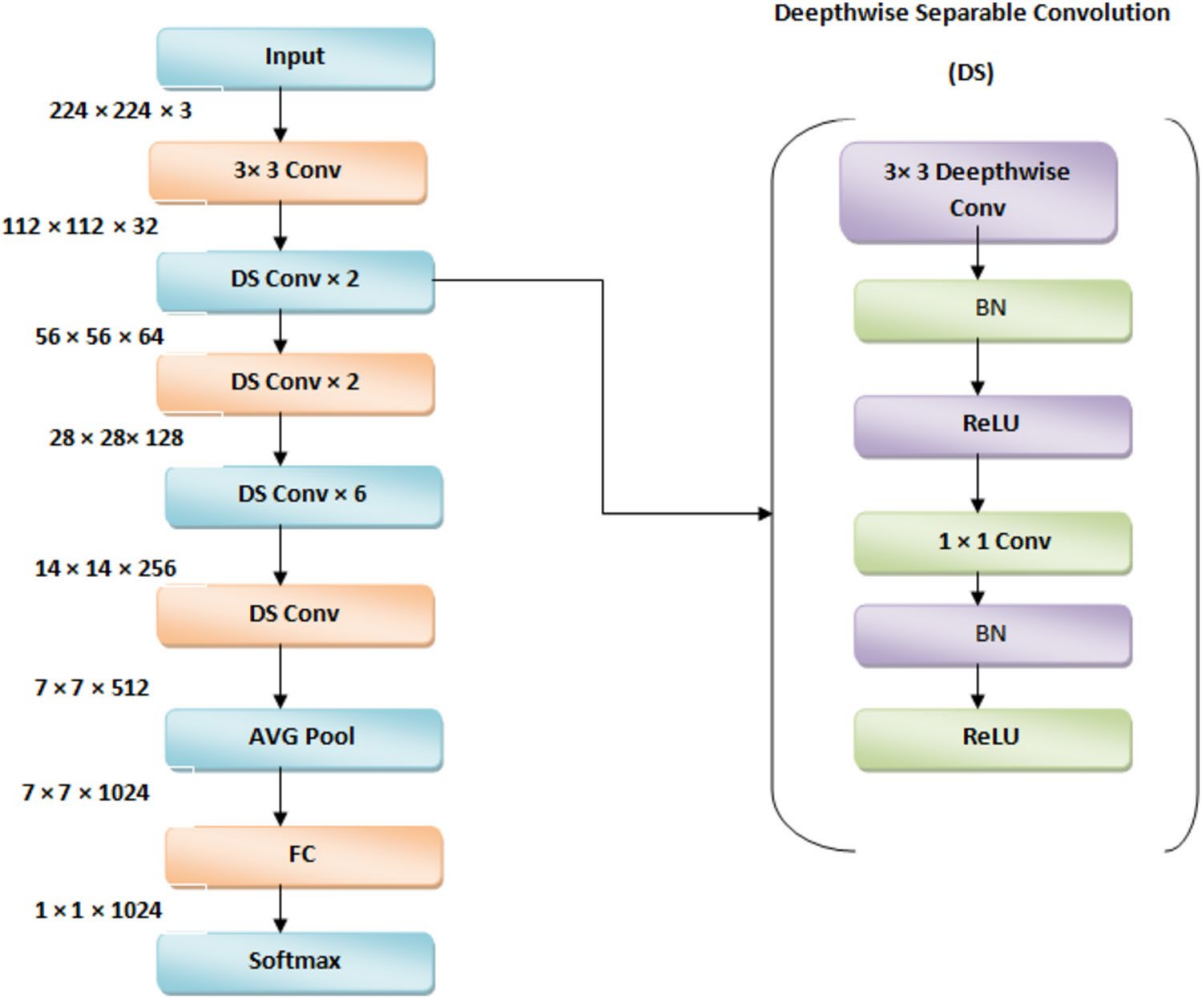
The main framework of MobileNet model.

To examine SAdagrad, we used two models: a simple CNN model and YOLO model. Figure 2 illustrates the proposed framework of CNN with learning transfer where Fig. 3 clarifies the main structure of Mobilnet model in details. This work introduces a new model for enhancing the behavior of adaptive optimizer Adagrad by combining the weight decay and fine-tuning techniques together while classifying the CRC dataset. It depends on the technique of transferring learning from a very complicated model on large dataset, such as ImageNet, to another new model on a different dataset. Figure 2 clarifies the step of transferring learning and how the fine- tuning technique works. Moreover, the coming sub-sections illustrate the steps of our proposed model in detail.

Second we apply SAdagrad optimizer on YOLO^44^ (You Only Look Once) classification model Object detection and classification algorithms can be divided into two types: one-step and two-step techniques. One-step approaches anticipate categories and limits for the whole image in a single instant, giving them a speed advantage over two-step algorithms. Two-step techniques, on the other hand, begin with the finding of significant areas followed by a categorization to determine whether or not an object was discovered in those areas. The existence of this preceding stage lowers the pace of the two-stage. YOLO model structure along with hyperparameter values tailored for training process (Input Size: 416 × 416 pixels, Activation Function: ReLU, Batch Normalization: applied after each convolution layer, Spatial Pooling: Max pooling layers to reduce spatial dimensions, Learning Rate: 0.001, Batch Size: 16, Number of Epochs: 50, Loss Function: combination of localization loss (mean squared error) and confidence loss (binary cross-entropy) weighted by λ parameters. Data preparation is a crucial step to make the data ready for network input^45^, Next section focus on these processes in details.

### Data preprocessing and data augmentation

The preprocessing stage is required to make sure that the data is in a format that the network can use to learn from and to enhance its performance. This paper employs preprocessing steps that are compatible with MobileNet model. Therefore, the following steps in the preprocessing phase that can be applied to the input data before feeding it into MobileNet. As the MobileNet model typically takes images of size 224 × 224 pixels as input, then all the images are resized to that standard size. The pixel values of the input images should be normalized to have zero mean and unit variance. This stage improves in the model's Convolutionergence during the training phase. Convolutionerting images to an appropriate format that the model can understand^46^ are a very vital step. Then, the data augmentation, which entails generating extra training examples by subjecting the original images to rotations, translations or flips, is employed. We apply color and spatial changes to each tissue. Images and binary masks undergo several spatial modification procedures. Random vertical and horizontal flips, as well as random zoom cropping, are applied to the photographs. Then, we use rotations of 90° and 180° to replicate the various orientations from a pathologist's perspective. We randomly adjust the brightness, contrast, and saturation of each slide, and then convert images to grayscale with varying probability. The performance of the network can be enhanced through data augmentation, which can also help avoid over fitting. The next three sub-sections explain the fine-tuning Technique, MobileNet structure, and The Weight Decay technique.

### Transfer learning using MobileNet

Fine-tuning represents one of the transfer learning approaches^47^ applied in deep learning to tune the Hyper parameter of a model that has been trained to suit an entirely novel task. In order to achieve the best performance of our model using SAdgrad optimizer there are many Hyper parameters which tuning in the training process like: learning rate and the number of neurons in each layer and finally the number of model’s layers. In order to do this tuning process we use one of the transfers learning approaches which are fine-tuning techniques.

Transfer learning is a machine learning approach that enhances learning of the specific problem by transmitting information (learning parameters) gained from a related source problem. There are several models that can transfer the learning parameters from pre-trained model to another model; this work uses CNN^48^ architecture called MobileNet^49^ as one of these transfer learning models. By employing the MobileNet model, which was previously trained on ImageNet dataset and its parameters were changed to meet our new training task, SAdagrad can be fine-tuned. To accomplish this, the MobileNet’s parameters were frozen up to a specific layer; and the remaining layers were subsequently trained using the desired data. In order to prevent the major changes to occur to the pre-trained parameters during fine-tuning with SAdagrad, the trained model is often beginning with the weights of the original one. Only the remaining layers of the trained model are taught with the new data at a lower learning rate with SAdagrad’s schedule and the frozen layers are not updated during training^50^.

To minimize the computational complexity, it makes use of depth wise separable Convolutionolutions. Furthermore, the network has numerous layers, including point wise Convolutionolutional layers for dimensionality expansion and depth wise Convolutionolutional layers to gather spatial information. For enhanced performance, these layers are adhering to batch normalization and ReLU activation routines^51^.By utilizing depth wise separable Convolutionolutions, MobileNet achieves a fair compromise between accuracy and efficiency. Therefore, it is appropriate for devices with limited resources, maintaining respectable recognition abilities for image categorization and object identification applications.

To avoid overfitting in the proposed model, the regularization technique (L2) as one of the popular weight decay technique is used. The next subsection focuses on regularization method.

### Avoid overfitting using the weight decay technique

Weight decay^52^ is a deep learning approach intended to avoid overfitting. It is also called regularization technique. It occurs when a model is too complicated and struggles to generalize to new untried data. The most popular regularization types are L1 and L2. These techniques add term called regularization term to the activation function that handle the overfitting issue in the training and testing processes as clarified in Eq. (7). Where w is the weight vector, AY is the model's actual outcome, while DY is the planned outcome and λ is the hyper parameter whose value has been tuned for improved outcomes is this one.

7 $$ {\text{Activation function}} = {\text{ difference }}\left( {{\text{AY}},{\text{ DY}}} \right) \, + \, \lambda \, *\sum \left| {\left| w \right|} \right|^{2} $$

Since L2 regularization drives the weights to decay near zero (but not completely zero). L2 regularization method usually referrers to the weight decay or ridge regression. During training for L2 regularization, a penalty term is introduced to the model's loss function. This penalty term incentivizes the model to maintain the minimal network weights. The total of the squared values of each weight in the model is multiplied by a regularization parameter.

The model is incentivized to disperse the weights over all the features rather than depending disproportionately on a small number of features by including this term in the loss function^53^. The next section shows the experiments and the evaluation of the experimental results.

### Evaluation metrics

This section presents and clarifies the outcomes of the proposed model with SAdagrad. Various metrics, including accuracy, precision, recall, and f1-score, to evaluate its performance is used to evaluate the performance of SAdagrad. This study also includes the precision and loss curves for both the training and the validation datasets. Common measures for assessing a classification model's performance include precision, recall, and F1-score^54^. The precision is defined as the ratio of true positive predictions (examples of positive outcomes that are accurately predicted) to all positive predictions (including TP and FP). In applications where FPs can have serious repercussions, as illustrated in Eq. (8), a high precision score indicates that the model has produced fewer erroneous positive predictions.

8 $$\text{Precision }=\frac{\text{TP}}{\text{TP}+\text{FP}}$$

The recall counts how many of the actual positive examples are correct forecasts. In applications where FN (i.e., failing to identify positive examples) have substantial repercussions, as shown in Eq. (9), a significant recall score implies that the model identified the majority of the positive cases in the dataset accurately.

9 $$\text{recall }=\frac{TP}{TP+FN}$$

The harmonic mean of precision and recall, or F1-score, finds a balance between the two metrics. It has a 0–1 scale, with 0 reflecting the poorest potential accuracy and 1 signifying perfect precision and recall. As illustrated in Eq. (10), F1-score is a helpful metric when precision and recall are taken into account simultaneously.

10 $$F1\text{ \_score }=\frac{2TP}{2TP+FP+FN}$$

### Experimental results

In this section, we display the comprehensive comes about and examinations gotten from our tests on CRC tissue picture classification. The essential objective of our consider is to assess the execution of the proposed show, especially in comparison to existing state-of-the-art approaches. The tests were conducted on a well-curate dataset of CRC tissue pictures, guaranteeing an assorted representation of obsessive conditions. The Experiments were carried out in a clearly defined setting using the Keras and TensorFlow frameworks to provide a reliable and uniform analytical setup. Hyper parameters, including learning rate default value for Adam and Adagrad optimizers and regard to proposed schedule for SAdagrad, batch size is 64 for all experiments, and we used three optimizers (SAdagrad, Adagrad and Adam), were meticulously tuned to strike a balance between model performance and training efficiency. In experiments micro average: this term refers to the average performance metric calculated across all classes in the dataset, giving equal weight to each class. Macro average: macro average calculates the average performance metric independently for each class and then averages these values to obtain an overall metric. It gives equal importance to each class, regardless of class imbalance. Table 1 illustrates the Accuracy, Precision, Recall, and F1-score of the experimental results of the proposed model using Adam optimizer on CRC dataset.

**Table 1:** The precision, recall, and F1-score of Adam.

CRC dataset				
Class	Precision	Recall	F1-score	Support
TUMOUR	1.00	1.00	1.00	10
STROMA	0.88	0.88	0.88	8
COMPLEX	0.88	1.00	0.93	7
LYMPHO	1.00	1.00	1.00	10
DEBRIS	0.80	0.80	0.80	5
MUCOSA	1.00	1.00	1.00	6
ADIPOSE	1.00	0.91	0.95	11
EMPTY	1.00	1.00	1.00	7
Micro average	0.95	0.95	0.95	64
Macro average	0.94	0.95	0.95	64
Weighted average	0.96	0.95	0.95	64
Sample AVG	095	0.95	0.95	64

**Figure 4:**
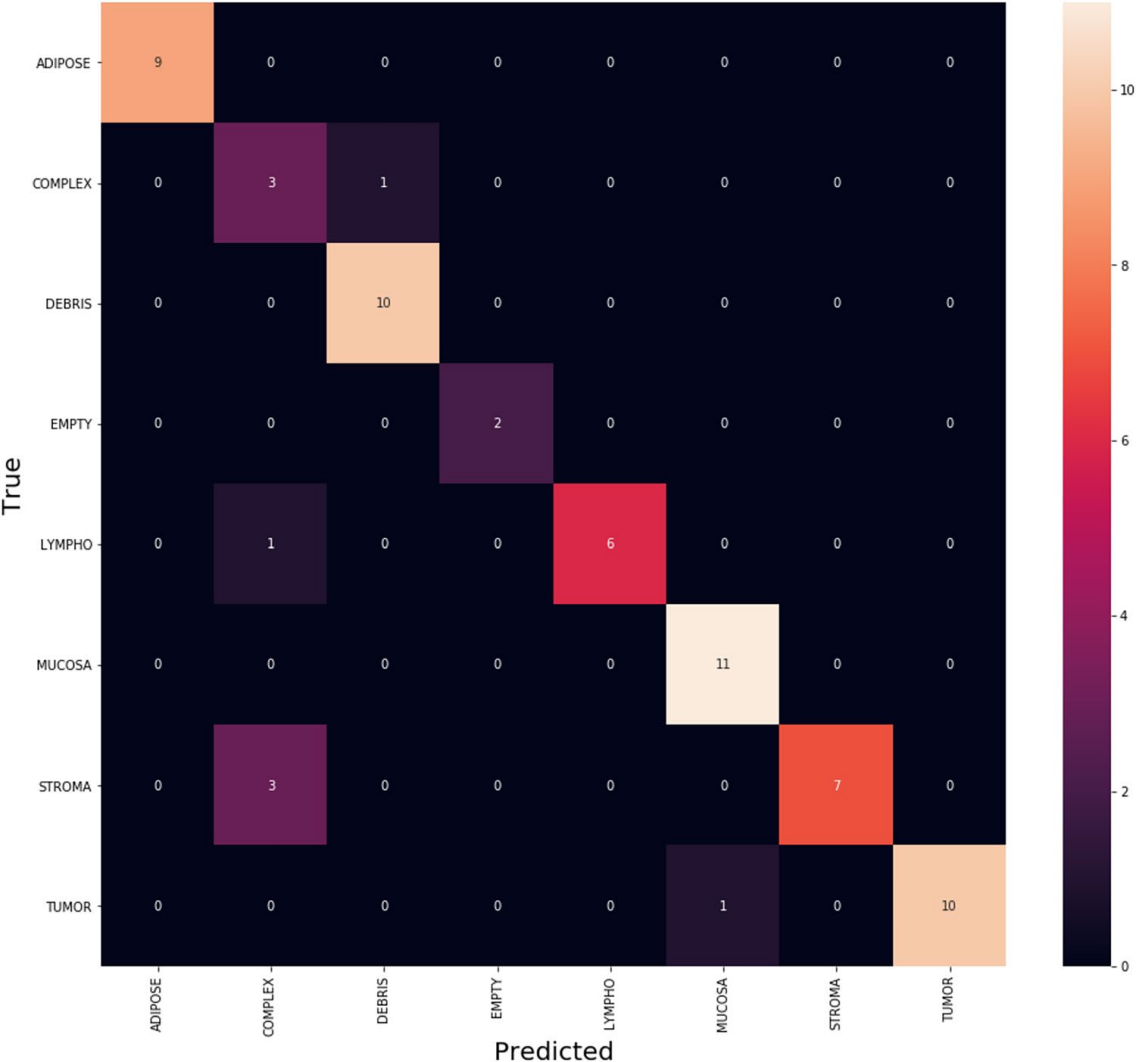
The confusion matrix of Adam.

In addition, the confusion matrices^55^ for the three algorithms Adam, Adagrad and SAdagrad. Figure 4 illustrates the confusion matrix of Adam optimizer which clarifies that Adam is getting confused in little number of classes with small values. By focus on some of higher values of negative classification are between these classes with same similarity. We found that classes with high confusion values are (MUCOSA, DEBRIS, and TUMOUR).

**Figure 5:**
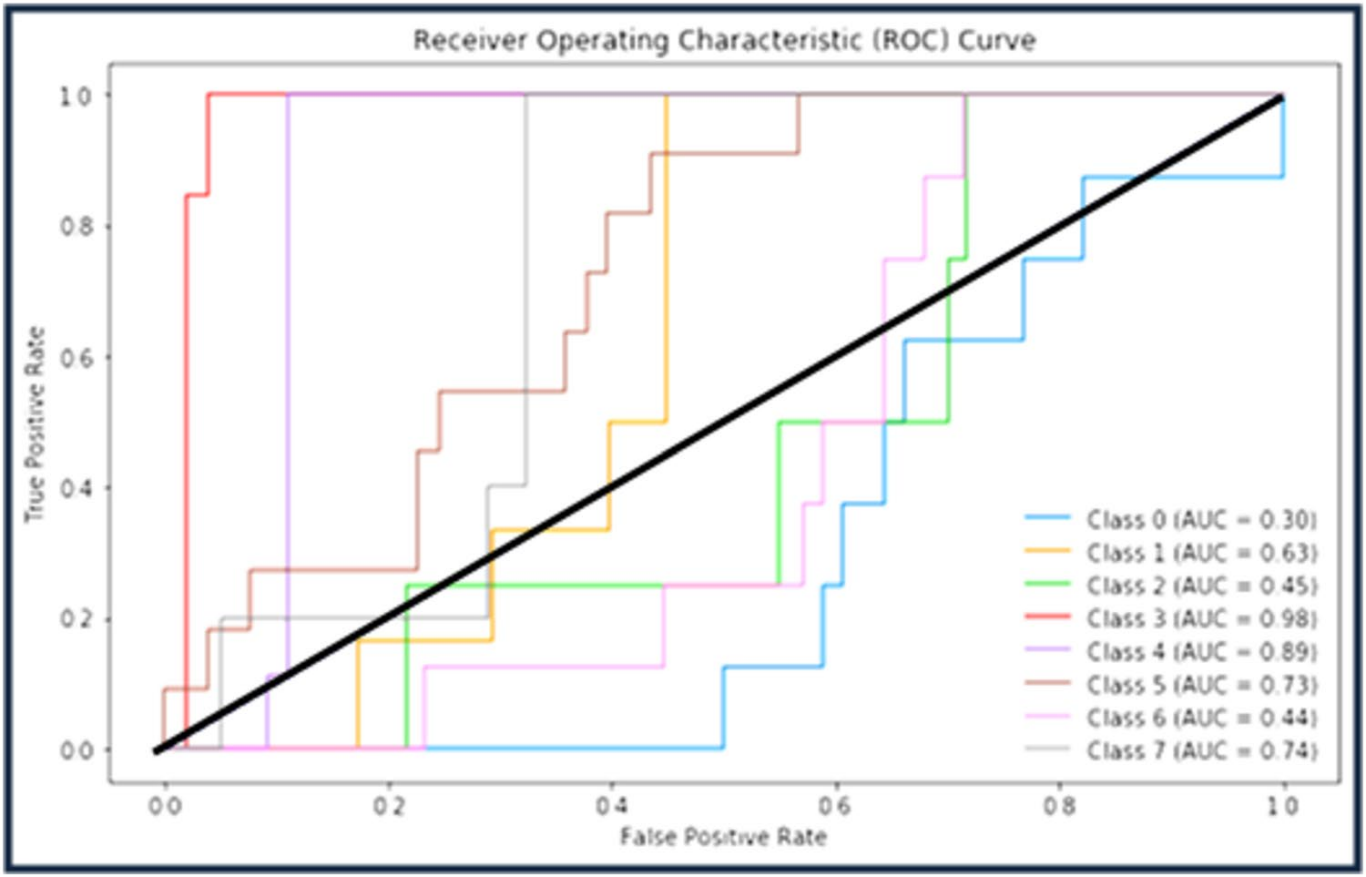
ROC curve for Adam.

Figure 5 clarifies ROC curve for Adam optimizer under the basic model. Where class 0 is TUMOUR tissue, class 1 is STROMA, class 2 is COMPLEX, class 3 is LYMPHO, class 4 is DEBRIS, class 5 is MUCOSA, class 6 is ADIPOSE, and class 8 is EMPTY*.*

Table 2 illustrates the Accuracy, Precision, Recall, and F1-score of the experimental results of the proposed model using Adagrad optimizer on CRC dataset.

**Table 2:** The precision, recall, and F1-score of Adagrad.

CRC dataset				
Class	Precision	Recall	F1-score	Support
TUMOUR	1.00	1.00	1.00	9
STROMA	0.43	0.75	0.55	4
COMPLEX	0.91	1.00	0.95	10
LYMPHO	1.00	1.00	1.00	2
DEBRIS	1.00	0.86	0.92	7
MUCOSA	0.92	1.00	0.96	11
ADIPOSE	1.00	0.70	0.82	10
EMPTY	1.00	0.91	0.95	11
Micro average	0.91	0.91	0.91	64
Macro average	0.91	0.90	0.89	64
Weighted average	0.94	0.92	0.92	64
Sample average	0.91	0.91	0.92	64

**Figure 6:**
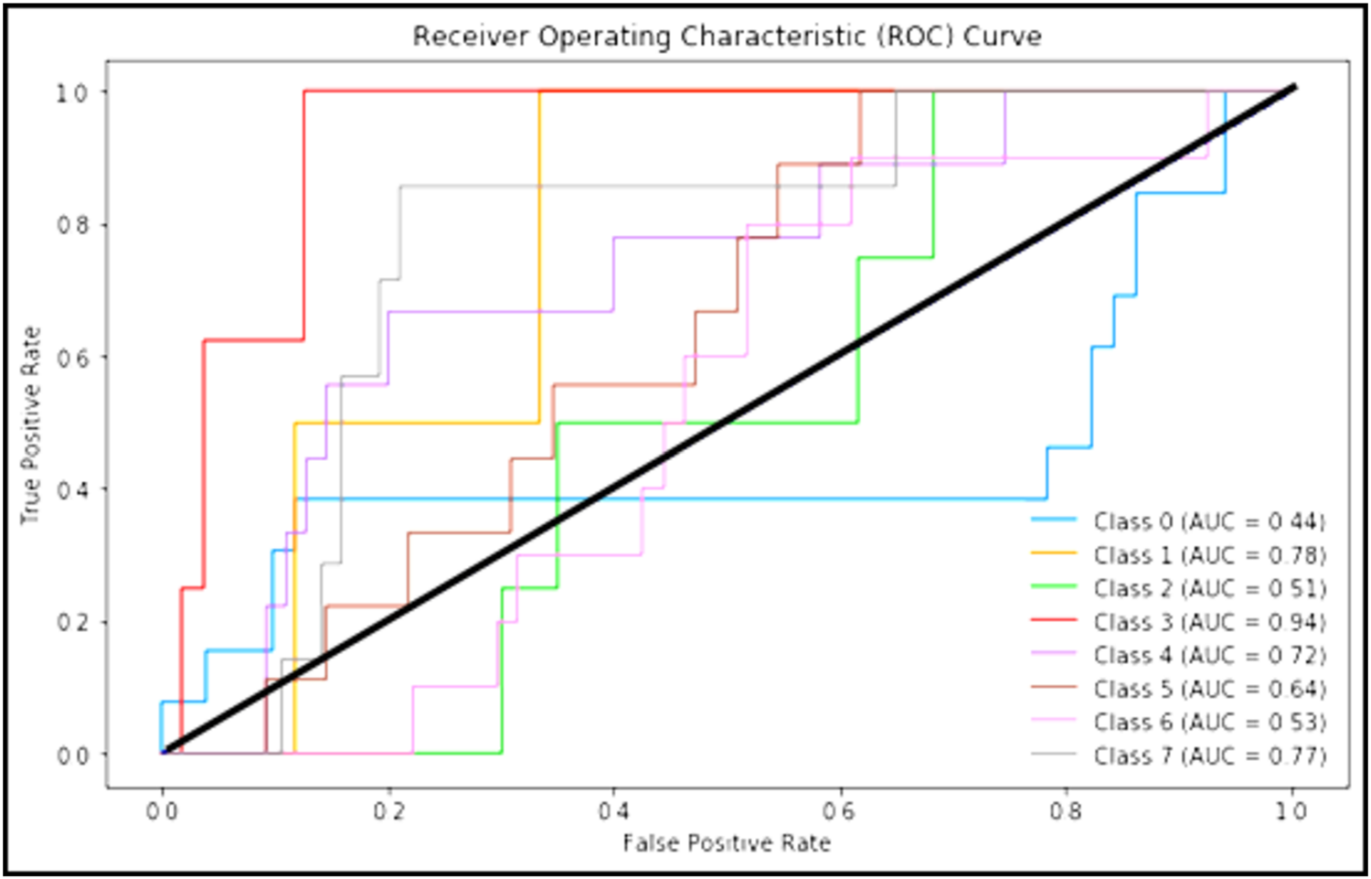
ROC curve for Adagrad.

Figure 6 clarifies the ROC curve for Adagrad optimizer under the basic model, where class 0 is TUMOUR tissue, class 1 is STROMA, class 2 is COMPLEX, class 3 is LYMPHO, class 4 is DEBRIS, class 5 is MUCOSA, class 6 is ADIPOSE, and class 8 is EMPTY.

**Figure 7:**
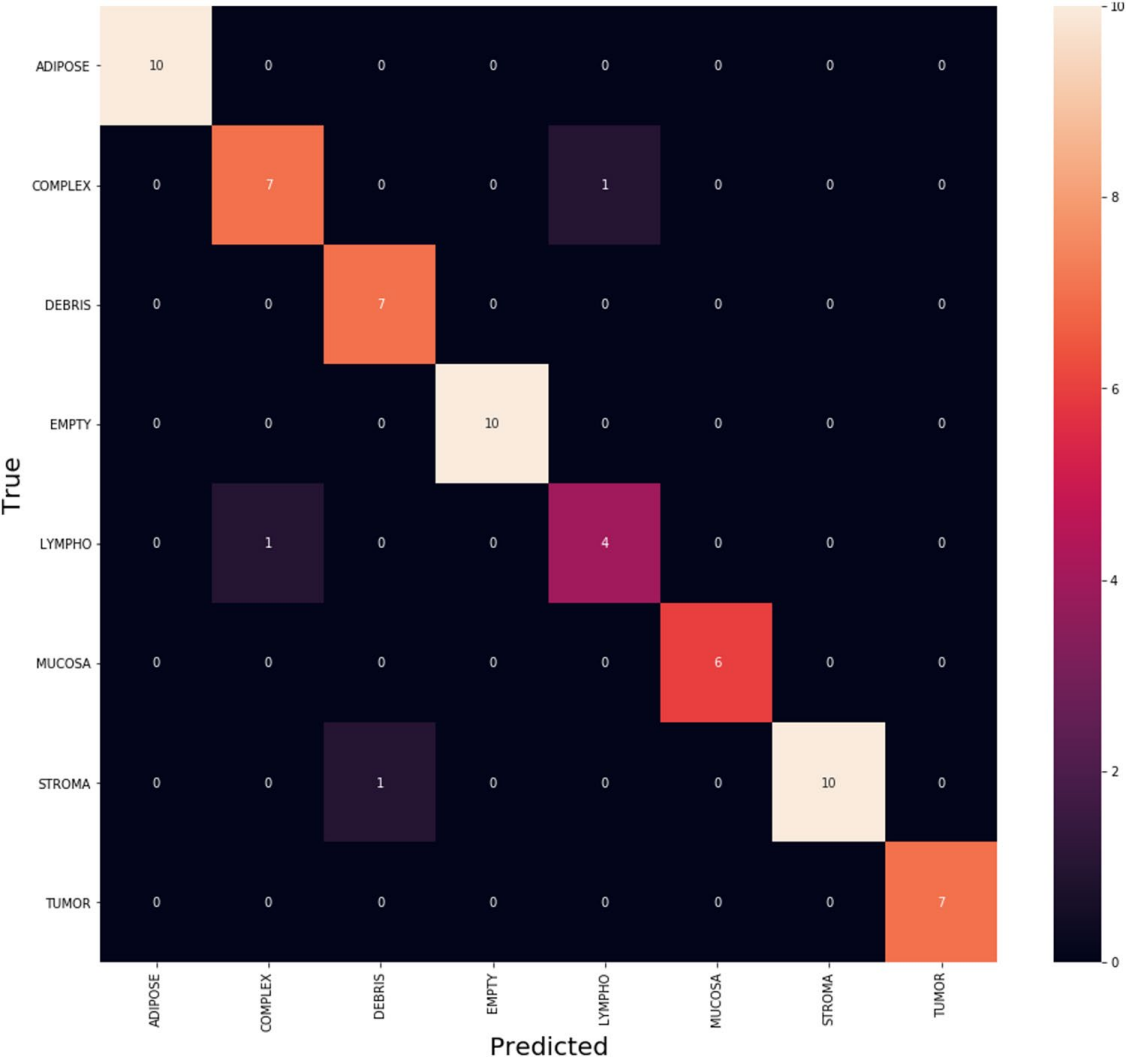
The confusion matrix of Adagrad.

Figure 7 clarifies the classes which Adagrad optimizer get confused in classification on CRC dataset optimizer which clarifies that Adam is getting confused in little number of classes with small values. By focus on some of higher values of negative classification are between these classes with same similarity. We found that classes with high confusion values are (STROMA, ADIPOSE and EMPTY).

Table 3 illustrates the Accuracy, Precision, Recall, and F1-score of the experimental results of the proposed model using the modified SAdagrad optimizer on CRC dataset.

**Table 3:** The precision, recall, and F1-score of SAdagrad.

CRC dataset				
Class	Precision	Recall	F1-score	Support
TUMOUR	1.00	1.00	1.00	7
STROMA	1.00	1.00	1.00	4
COMPLEX	1.00	0.83	0.91	6
LYMPHO	1.00	1.00	1.00	9
DEBRIS	1.00	1.00	1.00	2
MUCOSA	1.00	1.00	1.00	8
ADIPOSE	0.80	1.00	0.89	4
EMPTY	1.00	1.00	1.00	10
Micro average	0.96	0.96	0.97	64
Macro average	0.97	0.98	0.96	64
Weighted average	0.97	0.98	0.98	64
Sample average	0.98	0.98	0.98	64

**Figure 8:**
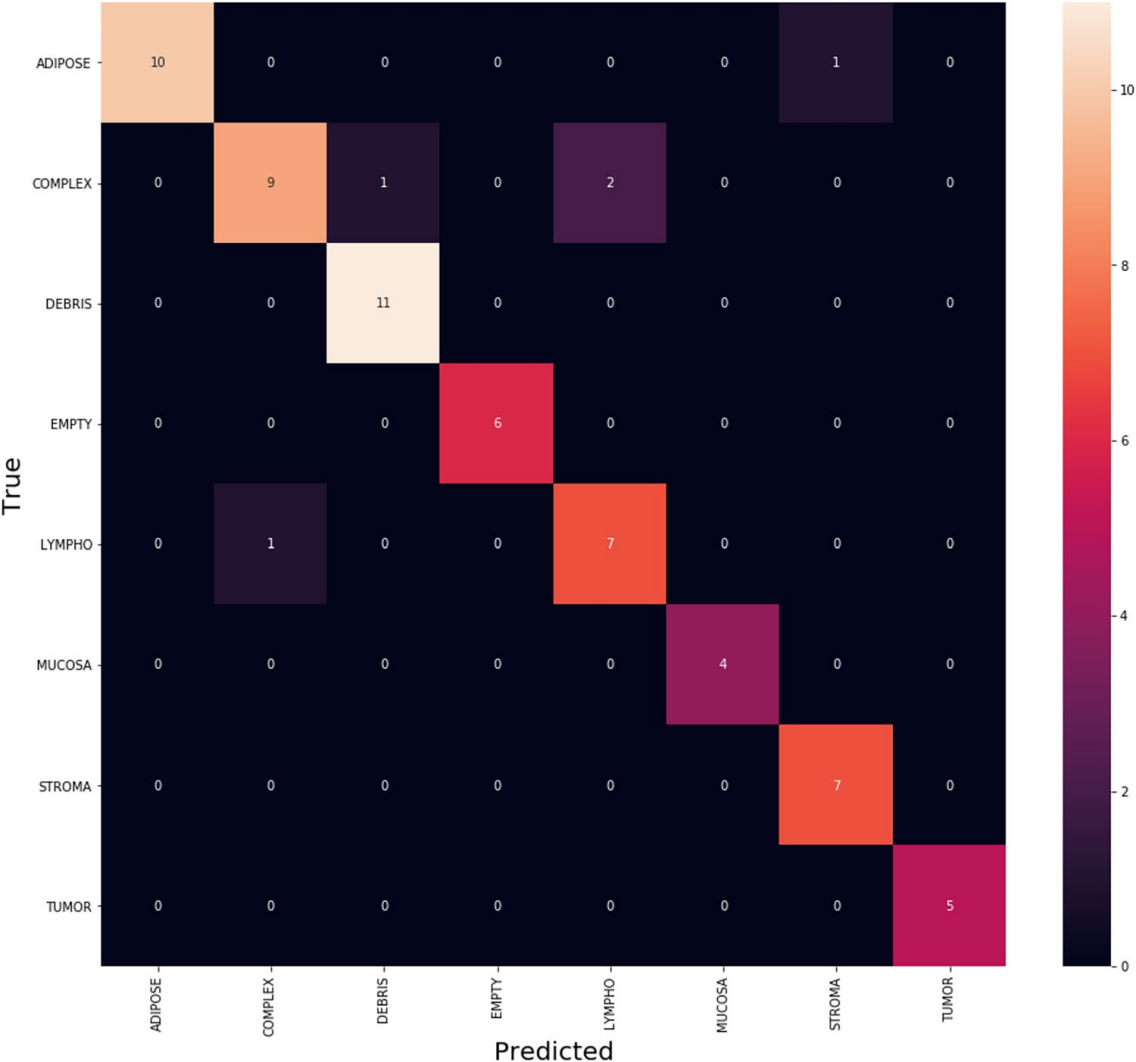
The confusion matrix of SAdagrad.

Figure 8 clarifies the classes which SAdagrad optimizer get confused in classification on CRC dataset which clarifies that Adam is getting confused in little number of classes with small values. By focus on some of higher values of negative classification are between these classes with same similarity. We found that classes with high confusion values are (COMPLEX, DEBRIS and ADIPOSE).

**Figure 9:**
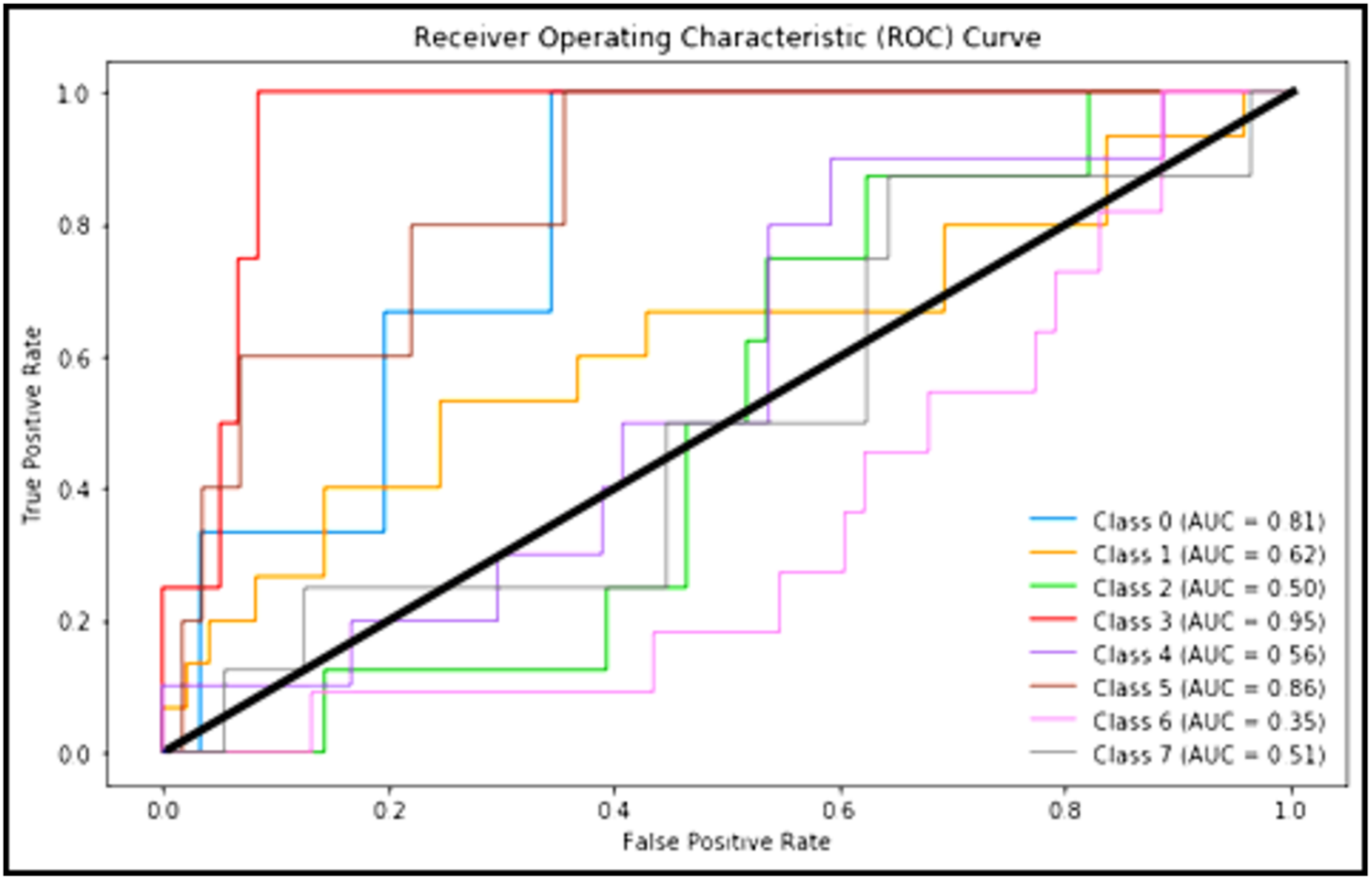
ROC curve for SAdagrad.

Figure 9 clarifies the ROC curve for SAdagrad optimizer under the basic model, where class 0 is TUMOUR tissue , class 1 is STROMA, class 2 is COMPLEX, class 3 is LYMPHO, class 4 is DEBRIS, class 5 is MUCOSA, class 6 is ADIPOSE, and class 8 is EMPTY.

**Figure 10:**
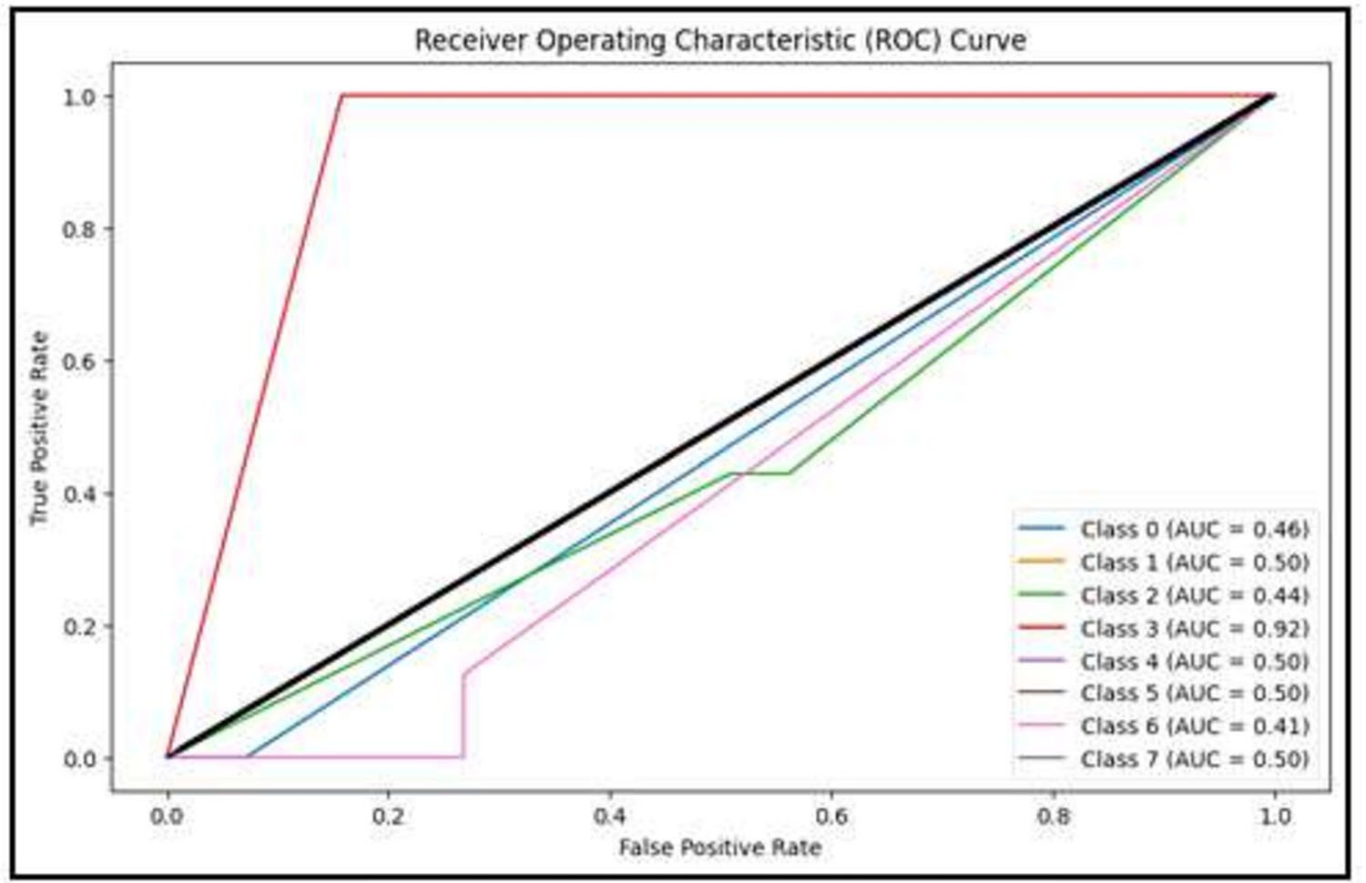
ROC curve for Adam.

**Figure 11:**
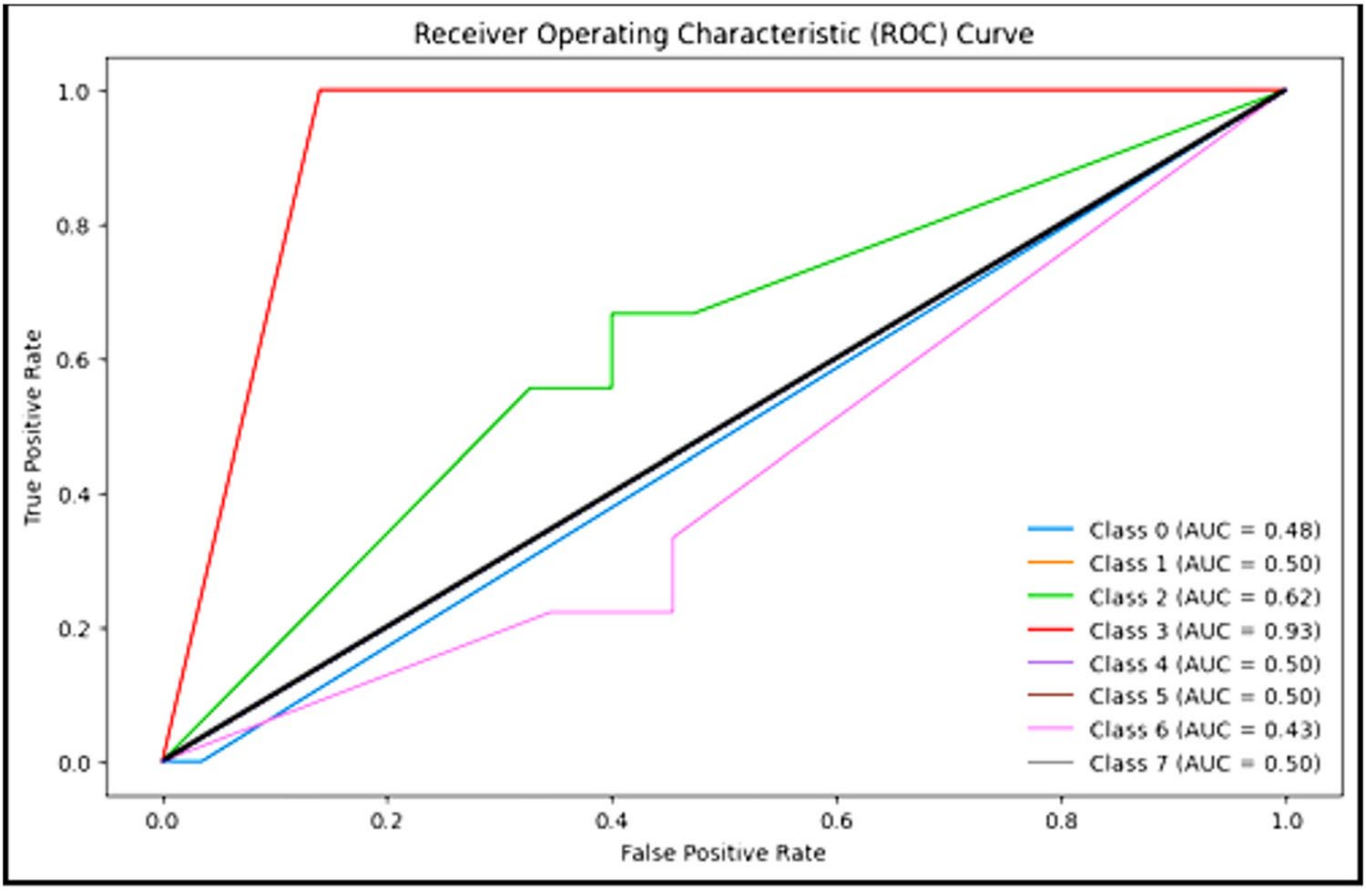
ROC curve for Adagrad.

**Figure 12:**
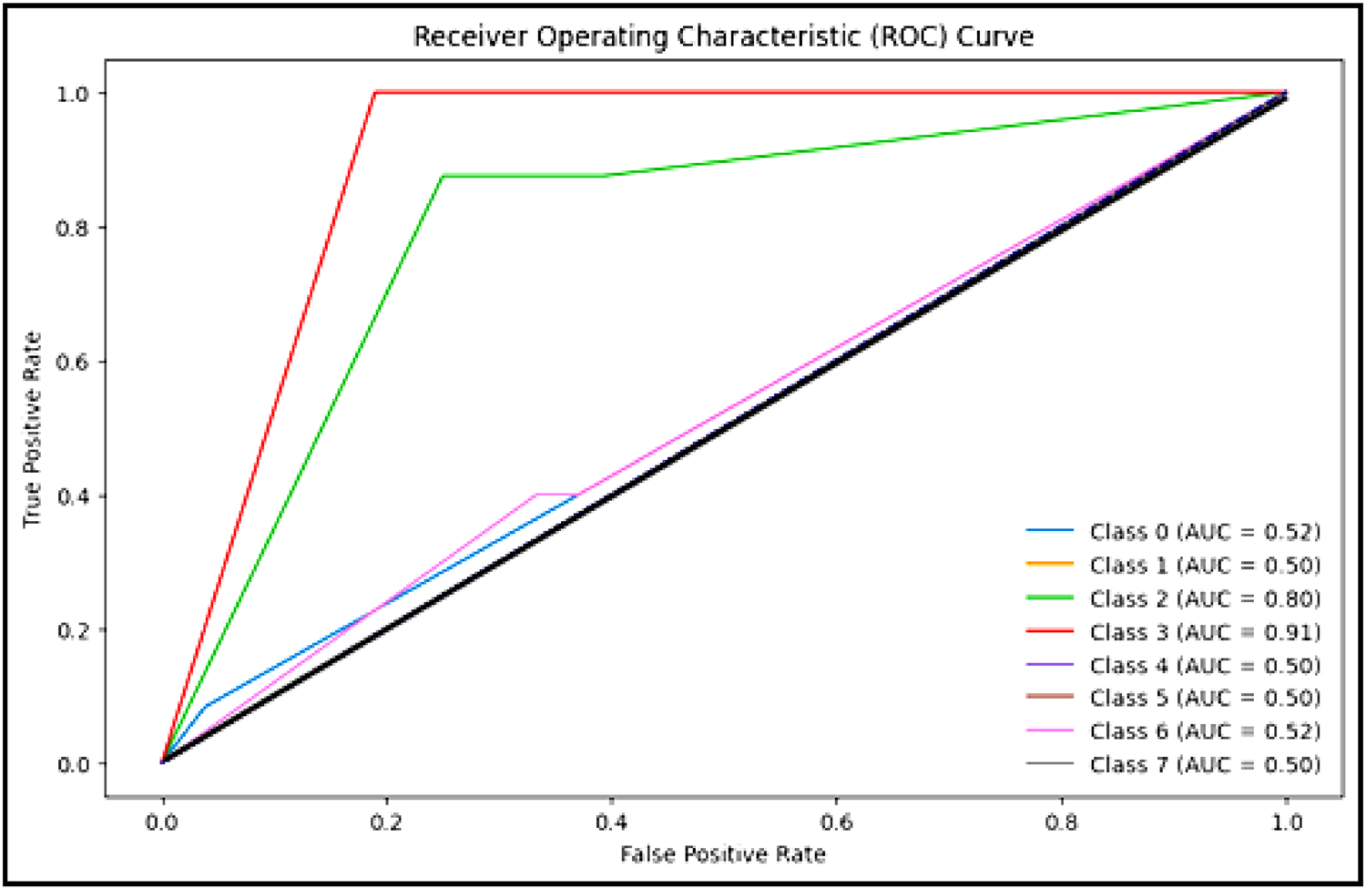
ROC curve for SAdagrad.

The following Figs. 10, 11 and 12 clarify the behavior of Adam, Adagrad and SAdagrad optimizers under YOLO model.

Figure 10 clarifies ROC curve for Adam optimizer under the YOLO model. Where class 0 is TUMOUR tissue, class 1 is STROMA, class 2 is COMPLEX, class 3 is LYMPHO, class 4 is DEBRIS, class 5 is MUCOSA, class 6 is ADIPOSE, and class 8 is EMPTY*.*

Figure 11 clarifies ROC curve for Adagrad optimizer under the YOLO model. Where class 0 is TUMOUR tissue, class 1 is STROMA, class 2 is COMPLEX, class 3 is LYMPHO, class 4 is DEBRIS, class 5 is MUCOSA, class 6 is ADIPOSE, and class 8 is EMPTY*.*

Figure 12 clarifies ROC curve for SAdagrad optimizer under the YOLO model. Where class 0 is TUMOUR tissue, class 1 is STROMA, class 2 is COMPLEX, class 3 is LYMPHO, class 4 is DEBRIS, class 5 is MUCOSA, class 6 is ADIPOSE, and class 8 is EMPTY*.*

Table 4 shows the comparative accuracy values for the proposed model using various Adam, Adagrad and SAdagrad optimizers.

**Table 4:** The accuracy of the three optimizers on the CRC dataset under the basic model.

Adam	Adagrad	SAdagrad
95%	92%	98%

Table 5 illustrates the accuracy of the proposed model using SAdagrad optimizer in comparison to earlier research on the same dataset. The proposed model remarkably outperforms the original publication and other works on this dataset, which significantly contributes to the field of CRC tissue categorization research. This work proposed that the combination of the fine- tuning technique and weight decay technique with SAdagrad on image identification for histopathology presents promising potential.

**Table 5:** Comparison between our models and previous literature.

Author	Year	Dataset	Model	Accuracy (%)
Kather et al.^15^	2016	CRC dataset	CNN models	87.4
Janowczyk^16^	2016		AlexNet, VGGNet and GoogLeNet	79
Wang^26^	2019		Deep learning model incorporating transfer learning	88
Cruz-Roa^20^	2014		CNN model	93
Rizalputri^22^	2020		K-nearest neighbor	82
Ohata^24^	2021		k-Nearest Neighbours, MLP, Random Forest, Naive Bayes, and SVM	92
Proposed model			CNN incorporating transfer learning with proposed SAdgrad optimizer	98

## Conclusion

The objective of this work is to introduce an enhanced version of Adagrad optimizer (SAdagrad) to avoid the drawbacks of Adagrad represented in tuning the learning rate values in each step in the training process. In Adam’s experiments optimizer while dynamically adjusting learning rates for each parameter, Adam accelerates convergence and enhances optimization performance. Its scale-invariant learning and regularization effects bolster robust optimization across diverse datasets and architectures. However, Adam's sensitivity to hyperparameters, particularly the learning rate, can lead to suboptimal convergence or instability during training. Moreover, its memory-intensive nature, stemming from maintaining two moving average vectors per parameter, can limit scalability in memory-constrained environments. Additionally, Adam may exhibit reduced robustness in the face of noisy or sparse gradients, potentially impacting optimization performance. These considerations underscore the importance of thoughtful hyperparameter tuning and careful application of Adam in deep learning workflows*.* In Adagrad’s experiments optimizer while it effective in many scenarios, faces several limitations that can hinder its performance in certain contexts. One significant drawback is its tendency to decay learning rates monotonically over time, particularly for frequently occurring features, which can lead to premature convergence or poor generalization. Additionally, Adagrad's accumulation of squared gradients individually for each parameter may result in overly aggressive updates for parameters associated with sparse features, diminishing optimization effectiveness. Furthermore, the algorithm's memory requirements can be prohibitive, as it necessitates storing a separate learning rate accumulator for each parameter, especially in models with a large number of parameters. Lastly, Adagrad's lack of momentum adaptation may limit its ability to navigate complex loss landscapes or escape from local minima efficiently. These limitations highlight the importance of considering alternative optimization strategies or enhancements tailored to specific optimization challenges which we improved in SAdagrad enhanced optimizer. In order to examine SAdagrad, CNN model is created which works based on two techniques. The first technique is fine-tuning, in which a pre-trained model is further trained on a new dataset with the goal of adapting it to the new task at hand. It focuses on using the learned features of the pre-trained model as a starting point and then updating the weights of the model during the training process to better fit the new data. The second technique is the weight decay technique. It is a deep learning approach intended to avoid overfitting; it is also called regularization technique. It takes place when a model is too complicated and struggles to generalize to new untried data. The CRC dataset has been employed to train the model. The experimental results presented in this paper demonstrate that the integration of these techniques with the SAdagrad optimizer has led to an improvement in the behavior of Adagrad, with 98% accuracy.

## Limitations and future directions

The current methodology represents a considerable step forward from prior incarnations of Adagrad optimizer that helps to avoid the main drawbacks of Adagrad of vanishing the value of learning rate for each iteration, However, the study’s introduce SAdgrad optimizer that show more efficient performance in training and testing process of CRC dataset and achieve high accuracy more than the others optimizers (Adam and Adagrad) under the same models, it may take cost more time in training process than Adam and Adagrad. In order to avoid this in the future it would be desirable to enhance the SAdagrad model's proficiency to detect more sophisticated and changing in the datasets. This includes investigating new hyperparametres values that adapt the leaning process of the model to increase the model's accuracy and effectiveness. Also, the SAdagrad optimizer could be integrated with the existing Nestrove technique as hybrid deep learning framework. Implement Nesterov to SAdagrad to accelerate its convergence in details: Nesterov Accelerated Gradient (NAG) is a modification of the momentum method that helps accelerate the convergence of optimization algorithms. By adjusting parameter updates based on an estimate of the future gradient, Nesterov momentum allows for faster convergence, especially in areas of the parameter space with large curvature. Here's how Nesterov acceleration speeds up the optimization process: improved gradient estimation: in standard momentum methods, the current gradient is used to estimate the direction of the update. However, this approach can lead to overshooting, especially in regions with high curvature. Nesterov momentum computes the gradient at a point slightly ahead in the direction of the momentum term. This "lookahead" helps provide a more accurate estimate of the gradient's direction, reducing oscillations and overshooting. Faster convergence: by incorporating information about the future gradient, Nesterov momentum accelerates convergence towards the minimum. The algorithm tends to take larger steps in directions with steep gradients while gradually slowing down as it approaches the minimum. This enables faster progress through the parameter space, leading to quicker convergence. Enhanced robustness: Nesterov acceleration improves the robustness of optimization algorithms by reducing the likelihood of getting stuck in local minima or saddle points. By providing a more accurate estimate of the gradient direction, Nesterov momentum helps the optimizer escape shallow local minima and navigate through regions with high curvature. Consistent momentum handling: unlike traditional momentum methods that may overshoot or oscillate around the minimum, Nesterov acceleration ensures smoother and more consistent updates. By adjusting the momentum term based on the lookahead gradient, the algorithm maintains stability and avoids erratic behavior during optimization. This implementation has an impact of capturing the speeding up advantages of momentum while permitting the pursuit to calm down as it approaches the optima, reducing the possibility of missing or exceeding it. The combination of these techniques can increase the speed of the optimizer and give it more stability in its performance.

## Data Availability

The datasets (CRC Dataset) used during the current study available in the following links respectively: https://www.kaggle.com/datasets/user322312312/kather-texture-2016-image-tiles-5000-1.
